# A systematic review of classification systems for pilonidal sinus

**DOI:** 10.1007/s10151-019-01988-x

**Published:** 2019-05-16

**Authors:** E. M. Beal, M. J. Lee, D. Hind, A. P. Wysocki, F. Yang, S. R. Brown

**Affiliations:** 1 University of Sheffield, The Innovation Centre, 217 Portobello, Sheffield, S1 4DP UK; 2 Department of General Surgery, Sheffield Teaching Hospitals, Sheffield, UK; 3 Department of Surgery, Logan Hospital, Meadowbrook, QLD Australia; 4 Griffith Health Centre, Griffith University Medical School, Gold Coast Campus, Griffith University, Queensland, Australia

**Keywords:** Pilonidal sinus disease, Classification system, Colorectal surgery

## Abstract

**Background:**

Pilonidal sinus disease (PSD) is a simple chronic inflammatory condition resulting from loose hairs forcibly inserted into vulnerable tissue in the natal cleft. It is an acquired disease with a slight familial tendency. There is no agreement on optimum treatment and the multitude of therapeutic options cannot be compared due to the lack of a universally adopted classification of the disease. The aim of our study was to perform a systematic review of the literature to determine how presentations of PSD are classified and reported.

**Methods:**

A systematic review of the English language literature was undertaken searching studies published after 1980.

**Results:**

Eight classification systems of PSD were identified. Most classification systems were based on anatomical pathology hypotheses. The location and number of sinuses were the main factors defining classification systems. No articles were retrieved that assessed the validity and/or reliability of the classification system employed. Furthermore, there was no evidence to suggest a correlation between prognosis outcome and subgroup.

**Conclusions:**

Based on the evidence available from the literature reviewed we have no recommendations regarding the use of the current classification of PSD. A well-recognised and practical classification system to guide clinical practice is required.

## Introduction

Pilonidal sinus disease (PSD) is a common condition affecting 26/100,000 of the general population, predominantly young, and employed males [1]. PSD is rarely self-limiting and, therefore, surgery is the mainstay of treatment. Many surgical methods are described including sinus/pit-based procedures, excision with open management, excision and midline closure, and excision with completely off midline flap repair or flap closure which crosses the midline. Adjuvant laser treatment and shaving are also described. Despite all these options, the recurrence may be as high as 60.4% at 24 months post-surgery [2]. In addition to recurrence, early wound complications such as infection and dehiscence are very common [3–5].

PSD is an umbrella term for a spectrum of abnormalities ranging from relatively asymptomatic simple midline pits or sinuses to complex chronically inflamed cavities with multiple fistulous tracks to treatment failure. Most patients present with chronic symptoms but a significant proportion present with an acute abscess. Different stages of the disease may be amenable to different treatment strategies. However, there is no universally adopted classification system for disease appearance.

## Classification

Clinical classification has a prognostic function. The Prognosis Research Strategy (PROGRESS) Group summarizes and proposes a stepwise approach to prognosis research. Ultimately this allows stratified treatment. In the treatment of PSD, the existing classification systems are not used in routine practice to guide treatment as clinicians generally select treatment methods in accordance with their own experience and training [2]. Previous literature has outlined the prevalence of PSD [6] and evaluated surgical techniques [7] with the use of a classification system, however, few studies exist evaluating the use of a classification system for PSD that informs the choice of treatment. Where a classification system is proposed, its predictive properties should be assessed. Properties commonly assessed in classification systems are internal consistency (i.e., ensuring the items being used are measuring the correct constructs that are being investigated), reliability, measurement error, content validity (including face validity), construct validity (including structural validity, hypotheses testing and cross-cultural validity), criterion validity (i.e., the extent to which a measure is related to an outcome), responsiveness, and interpretability as per the COnsensus-based Standards for the selection of health Measurement Instruments group (COSMIN) [8]. This highlights the need for an accessible classification system that will allow the informed choice of treatment.

The aim of this systematic review was to provide an overview of published classification systems for PSD and to summarise any analyses of the reliability and validity of each system.

## Materials and methods

**Fig. 1:**
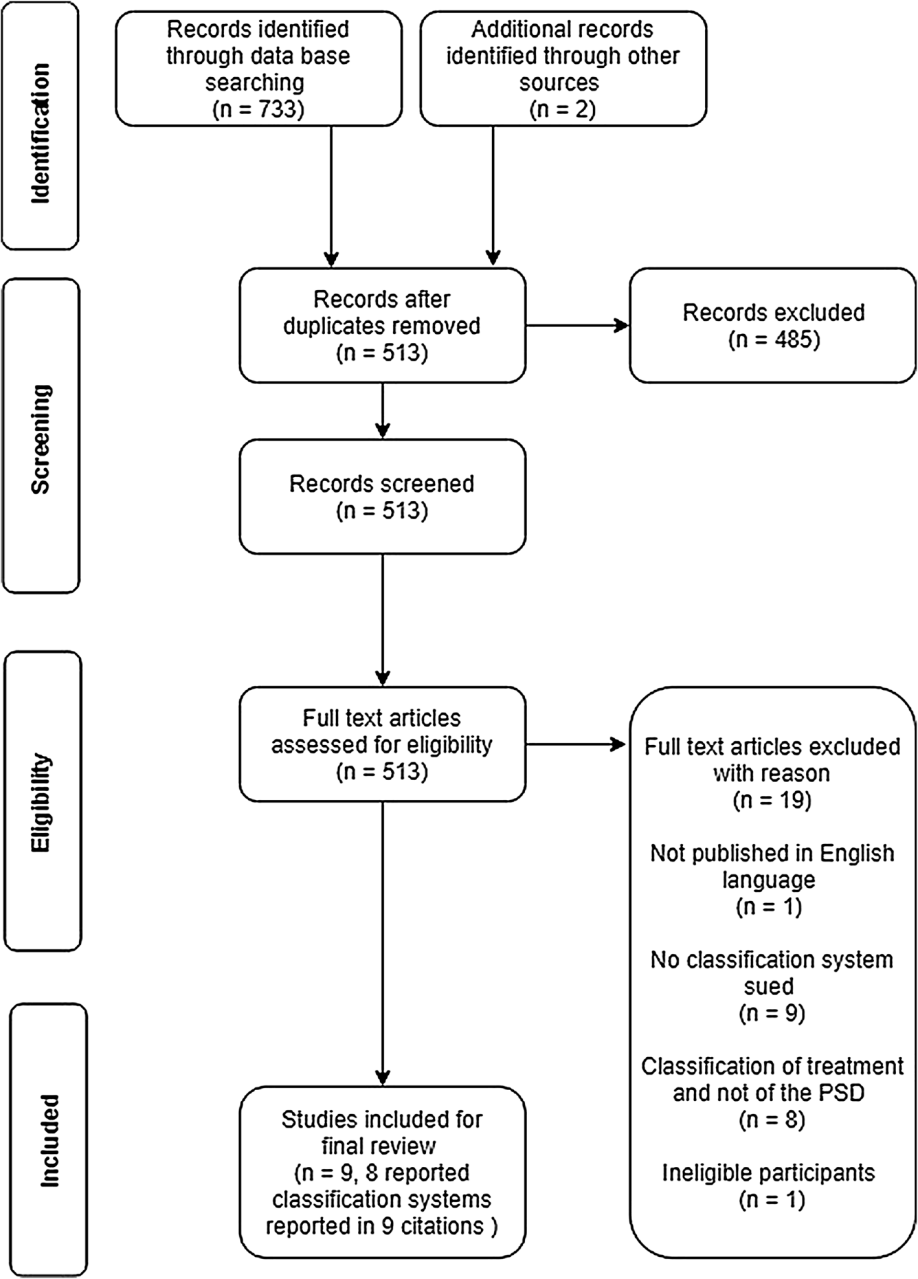
PRISMA flowchart

This systematic review of PSD classifications was registered with PROSPERO (Registration number: CRD42018111767). It is reported in accordance with the preferred reporting items for systematic reviews and meta-analysis (PRISMA) guidelines (for PRISMA flowchart see Fig. 1) [9]. The quality of the methods used in each study was assessed against the Consensus-based standards for the selection of health status Measurement Instruments (COSMIN) guidelines [8].

### Eligibility criteria

All original studies investigating the ways in which to classify the presentation of PSD were eligible for inclusion. This review focused exclusively on the classification systems used to describe PSD rather than therapeutic interventions. Systematic reviews, randomized controlled trials, observational studies (such as cohort studies), case–control studies and cross-sectional studies, could be included, when they described details of ways in which to classify PSD. Theoretical articles and empirical studies were also included. These included letters to the editor, reports or conference abstracts which proposed classification systems of PSD. Empirical work included case reports and primary research involving individual patients in which the reliability and validity of the classification system might be formally tested. Articles published after 1980 and in English were eligible for inclusion in the systematic review. Articles were excluded when PSD was present in a body region other than the natal cleft, were not written in the English language or did not specifically describe a classification system.

### Search and information sources

A search of the online databases MEDLINE and EMBASE was completed using keywords to search for articles published after January 1980. The MeSH search terms and keywords were “pilonidal disease”, “pilonidal sinus”, “SPSD”, “jeep disease” combined with “classification” or “classify” or “system” or “instrument” or “type” or “prognosis” or “predict” (sample search strategy is presented in “Appendix A”). A basic search of Google Scholar was also conducted. Furthermore, experts in the field (SRB, APW) were consulted for signposting to other relevant literature.

#### Study selection

Studies were screened for eligibility against the above criteria by two reviewers (EMB and FY). Where there was conflict in the assessment this was resolved by DH. Full texts were retrieved for the screening of the eligible studies. If a study was excluded, reasons were recorded. In cases where abstracts met inclusion criteria and a full text version of the study could not be retrieved the authors were contacted requesting further information.

### Data collection

The articles identified were included for full text review if they clearly indicated in the abstract or in the title that they had employed the use of or had proposed a classification system or diagnostic tool for PSD. These full text articles were then reviewed to ascertain whether a classification system had been created.

### Data extraction

The data items that were collected were study identification number (the number was generated by the initial of titles), citation (author, year of publication and countries where studies were conducted), study design, patients (number of different groups), the classification systems, the number of citation and the prognosis outcome (primary healing, functional recovery time, wound healing time, recurrence). Two reviewers (FY and EMB) recorded data independently and any conflicts or variations that arose were then discussed with a third reviewer (DH).

## Results

### Study selection

**Fig. 2:**
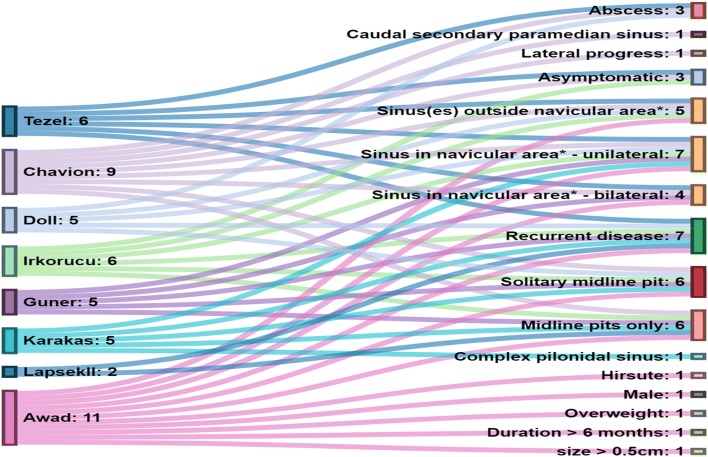
Alluvial diagram of classification systems. *Definition for navicular area: When the buttocks are pushed together, the outer lines of contact represent the lateral edges of the natal cleft. Its inferior extent is the posterior border of the anal triangle, which has its tip at the apex of the coccyx and its base between the ischial tuberosities [17]

Seven hundred and thirty-three records were screened for eligibility and 512 were assessed for eligibility. After the titles and abstracts were screened, a total of 27 records were assessed by full-text review and nine were included (Fig. 2). Articles were excluded when PSD was present in areas other than the natal cleft [9, 10]. Of the articles included, eight presented novel systems. One abstract was included even though the full text article could not be retrieved [11]. After conferring, it was decided to retain this study as the abstract detailed the classification system used which complied with the inclusion criteria. Two articles referred to the same classification system [12, 13]. One of these studies was included after consensus agreement. Among the papers included there were three letters to the editor. The remaining three studies presented longitudinal data on subgroup outcomes and had employed a classification system. One paper was a theoretical description [14].

## Synthesis of results

### Components of the classification systems

**Fig. 3:**
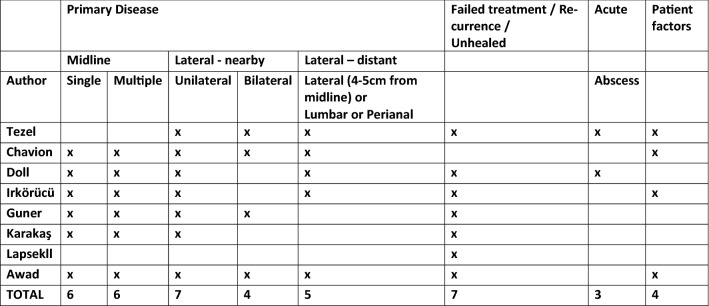
Classification systems: dimensions

Classification can be viewed as a logical process of sorting complex data into groups using shared features. The classification systems included produced overlapping dimensions as shown in Fig. 3. These were; classification due to location of sinus (although varying terminology was used) (*n* = 8) [11, 12, 14–19]; recurrent disease as a single dimension (*n* = 7) [11, 12, 14–19]; total number of pits/sinuses and tracks present (*n* = 4) [15, 16, 18, 19]; presence of abscess or treatment elements such as the need for drainage (*n* = 3) [14, 15, 17]; the size of the sinus or lesion (*n* = 2) [15, 18].

Three of the classification systems [12, 15, 17] included an asymptomatic sinus as a classification item in their systems. One classification system was based almost entirely on measurements (to anus, vertical and transverse disease extent) [14]. Only one classification system recorded patient as well as sinus characteristics [18]. This was the most detailed classification system, employing a scoring system based on the following factors: patient characteristics (hirsute or not, weight, sex), sinus characteristics (location, size, recurrence) and duration of symptoms.

Location of the sinus was present in all classification systems, although the terminology varied. Tezel’s [17] classification was based on the navicular area concept which was defined as “the extent of the natal cleft described by its lateral edges and posterior extent” with the patient in jack-knife position. Sinus openings within and outside the navicular area belong to different subgroups. Awad [18] classified location as either a sinus present in the midline or convex side. Irkörücü [12] defined the region as one or both sides of the natal cleft. Lapsekili [11] classified the areas as primary pits within a 1 cm area lateral to the intergluteal line, pits outside this area, pits under the imaginary line parallel to the anal canal in between the anal canal and coccyx. Guner’s [16] system classified location as midline and or lateral extension on one or both sides. Karakaş [19] divided this region into intergluteal sulcus, gluteal region, lumber region and perianal region. Chavoin [15] reported pits occurring in the midline area but did not classify them based on specific location. The classification systems of Chavoin [15] and Tezel [17] described symptoms and abscess formation.

In addition to the location of the sinus, Guner [16], Karakaş [19] and Chavoin [15] established that a single pit/sinus within the intergluteal sulcus could be classified as a separate category, as this represented mild disease that could be treated differently from multiple pits/sinuses. All classification systems, with the exception of Chavoin’s [15], referred to treatment failure (e.g., recurrent disease) as a separate category because the varied presentations affected complex treatment choices.

### Using a classification system to predict severity

Only two groups explored the use of a classification system in defining severity [17, 19]. The system coined by Guner [16] found a correlation between symptom duration and disease severity. However, this group did not compare the outcomes between disease stages. Awad’s [18] scoring system allowed patients to be divided into three groups according to the score. Treatment was then informed by the authors’ classification. The patient’s hospital stay had a positive correlation with their score and time for wound healing had a negative correlation likely related to the choice of procedure (see below). One further publication classified PSD into mild and severe and indicated what treatments should be performed for each [17]. However, this was not included in the analysis as the author did not describe the differences between a mild and severe presentation of PSD.

### Using a classification system to guide intervention

Tezel [17], Irkörücü [12, 13], Guner [16] and Chavoin [16] recommended that the management or treatment of PSD should be informed by a classification system. Tezel [17] proposed Bascom Cleft Lift for all elective symptomatic patients (types III–V). Irkörücü [13] proposed multiple procedures for each pilonidal disease type. Guner [16] grouped PSD into five stages. Pit picking was recommended if there were only 1 or 2 midline sinuses, otherwise the Bascom Cleft Lift was used for unilateral disease and the Limberg flap for bilateral disease. In Awad’s [18] scoring system those with low scores were managed by excision and healing by secondary intent, those with intermediate scores underwent excision and tension-free midline closure while these with highest scores underwent excision and unilateral or bilateral rotation flaps.

### Using a classification system to predict outcome

Three of the articles were discursive letters to the editor and did not present empirical observations of the outcomes obtained by varying sub groups [12, 17, 19]. Furthermore, Lapsekili [11] presented only cross-sectional observational data. The article by Awad et al. [18] provided longitudinal data on outcomes by categories of patients but did not assess whether the classification items caused varying outcomes for varying severities—3 months recurrence for all procedures and types was 2%. Guner and colleagues [16] highlighted a correlation between disease subgroups and the duration of symptoms. There was no correlation explained in the results and tables with only descriptive statistics outcomes shown, such as medians and ranges. Quinodzo [15] using Chavoin’s classification system outlined the surgical methods with the shortest recovery time and which surgical methods were more effective with decreased pain scores and shorter recovery time. However, the study measured the outcomes of the whole population rather than comparing the prognosis outcomes according to different subgroups. There were no articles that directly evaluated the prognosis of different subgroups as categorised by the proposed classification systems.

### Validation of classification systems

None of the retained studies included analyses to demonstrate the reliability or predictive validity of the proposed classification system. No articles directly evaluated the use of a classification system to inform treatment. Furthermore, none of the classification systems found were subjected to tests of reliability and validity. Therefore, critical appraisal using the COSMIN checklist [8] was not feasible. In addition, none of the articles evaluated the concurrent criterion validity. Similarly, construct validity and content validity were not assessed in any of the articles.

## Discussion

There are three potential roles of a classification system. Two are clinical; predicting prognosis and guiding treatment and the third is primarily for research purposes, allowing more precise comparative studies to be carried out. Our systematic review of classification systems for PSD found eight classification systems, none of which have been vigorously assessed for their prognostic characteristics, been adopted to guide management or been used routinely in surgical practice or comparative trials. All articles found used judgmental methodology to develop their classification systems. They identified homogeneous categories based on the experience of the investigators i.e., based on researcher practice and observation. In all the articles the cassification was mainly used to select patients for different procedures ranging from pit picking procedures for the lowers stages to flap closures for the most advanced stages of disease. However, no article provided analyses to demonstrate reliability or predictive criterion validity. Three articles list outcomes according to stage without a formal analysis [15, 16, 18]. However, for these articles different treatments had been selected for each subgroup, meaning it was impossible to distinguish the effect of the treatment or classification system from the prognosis of the condition. Despite these drawbacks this review gives some insight into what components may create a classification that is clinically useful and statistically valid.

Of the included articles almost all used anatomical position of pits/sinuses and secondary/lateral extensions (*n* = 7).

Position is likely to be important in defining complex and difficult to manage disease. For instance, proximity to the anus is crucial because it impacts on healing [20] and is likely to influence surgical management for 47% (52/112) of surgeons in a recent survey of clinical practice [21].

Midline disease may consist of tiny openings (pits) or the classical 2–3 mm openings (sinuses). The size of a sinus or lesion was mentioned in two systems included in the review. Undoubtedly there are a group of patients diagnosed with pilonidal disease who present with a long deep midline wound [20]. These types of patients, usually male, are certainly inappropriate for some surgical interventions (e.g., pit picking) and may respond differently to different surgical strategies. While some patients have only one midline opening, the implication of the number of sinuses/pits (e.g., less than or more than 5) is not clear but a high number may dissuade some surgeons from performing a minimally invasive procedure. However, the number of midline sinuses did not matter to 51% of survey respondents and the distance between highest and lowest sinuses did not matter to 55% of respondents [21].

A pilonidal sinus requires a midline site of hair entry (midline primary pit or sinus). Often there is an underlying cavity (originally termed “cyst”) which may discharge to the skin surface. This represents a lateral or secondary opening. This may present as cavity or healed scar which may be bilateral and is usually cephalad to the highest sinus [20]. Presence of lateral extension is part of seven of the eight classifications surveyed, weather within or outside the navicular area, and is considered as advanced stage and indication for closure with large flaps [7, 15–17]. In the survey only 37% of clinicians felt that the presence of a secondary extension did not affect their management while the majority felt that this had important treatment implications weather > 2–4 cm form midline (42%) bilateral (10%) or multiple (12%) [21]. The location of secondary extensions may also affect the type of surgery performed e.g., inferior (below coccyx) or superior (lumbar region) although 53% of survey respondents reported that it location below the tip of the coccyx had no treatment implications.

While “recurrences” are all different (e.g., depending on prior surgical history), what constitutes recurrent disease remains to be surgically defined. As some procedures are contraindicated (e.g., pit picking), failure of definitive management needs to be included as a separate category. What distinguishes an unhealed surgical wound from disease recurrence remains to be defined and it is not clear whether failure of definitive surgical management should be managed in the same way as primary PSD [20]. When asked if a surgical site which has not healed within 3 months be included in a proposed staging system 54% felt that it should but only on its own while 24% felt that it should be classed in the same category as recurrence [21].

Other important indicators are symptoms as they are important factors influencing decision-making about the type of treatment to use (surgical or non-surgical management). The absence of symptoms should not be considered in a morphological classification system because even morphologically extensive disease can be asymptomatic [20]. The presence of an abscess or need for drainage was included in two classification systems reviewed whilst others dealt specifically with chronic disease [15, 17]. Local oedema in the presence of an abscess makes assessment of midline disease difficult and the timing and nature of the chosen intervention will differ for acute vs chronic symptoms. The majority of abscesses point away from the midline and the resultant scar would then represent lateral/secondary disease extent in a chronic pilonidal sinus. Therefore, it is suggested that leaving acute pilonidal abscess out of classification would also reduce complexity.

Patient characteristics (*n* = 1) [18] such as hair and skin type, body weight, sex, ethnicity and even depth of natal cleft may influence prognosis but inclusion in any classification necessarily adds to complexity and is likely to detract from clinical utility.

A review is only as reliable as the literature upon which it is based; a limitation of this review is that half of the included papers retained were letters and small observational studies rather than large-scale experimental studies. In addition, none of the articles met the basic criteria so that their quality could not be evaluated using the COSMIN system [8]. The review, however, does highlight the need for further research into this area and evidences the dearth of clear universally accepted guidance for the classification of PSD. This paper is also the only review of the classification systems for PSD that the authors are aware of and, therefore, gives an overview of the classification systems currently in use.

## Future research

Various experts in the field have highlighted the need for a universally acceptable classification system [21]. Without such a system comparative trials have an unacceptable potential for selection bias. Bayhan et al. [17] is an example where the authors admit to the weakness of their work due to a lack of a classification system. This may explain to some extent the multitude of interventions for PSD, with many studies reporting that their authors’ favoured procedure has exceptionally low levels of recurrence [17]. For the average surgeon, the literature is bewildering and it is likely many surgeons simply continue to practice a procedure they are familiar with.

It is possible that a suitably pragmatic classification system could be integrated into clinical practice to support treatment decisions and the counselling of patients on likely outcomes. Such a system should be simple to use, reflect clinical practice and be meaningful in terms of prognostication. Commonly utilised examples outside cancer surgery do exist (e.g., Goligher’s system for haemorrhoids [22], and Park’s system for fistula in ano [23] but few to date have undergone rigorous validity testing. The development of such a system is one component of an ongoing United Kingdom cohort trial on pilonidal disease [24]. Key elements would include variety of midline openings, degree of secondary extent, extent below the level of the coccyx and treatment failure.

## Consensus statement

Based upon the available data, it is not possible to recommend any system to stratify severity of PSD or guide selection of treatment. Work to define a valid and reliable classification tool should be a priority. This will allow surgeons to confidently stratify disease and offer the most appropriate treatments.

## Appendix A

Search history (15 searches) (MEDLINE via Ovid).

**Table:**

Searches	Results
1. Pilonidal sinus/	1768
2. Pilonidal sinus.mp.	1985
3. Pilonidal disease.mp.	463
4. SPSD.mp.	34
5. jeep disease.mp.	5
6. or/1-5	2050
7. Classification/	9755
8. classif$.mp.	1,012,390
9. system$.mp.	3,930,768
10. instrument$.mp.	839,229
11. typ$.mp.	3,100,218
12. prognos$.mp.	759,349
13. predict$.mp	1,439,158
14. or/7-13	8,938,510
15. 6 and 14	289
Result	489

Search history (15 searches) (EMBASE via Ovid).

**Table:**

Searches	Results
1. Pilonidal sinus/	2038
2. Pilonidal sinus.mp.	2098
3. Pilonidal disease.mp.	522
4. SPSD.mp.	46
5. jeep disease.mp.	5
6. or/1-5	2146
7. Classification/	311,576
8. classif$.mp.	1,033,086
9. system$.mp.	6,207,585
10. instrument$.mp.	552,605
11. typ$.mp.	3,652,745
12. prognos$.mp.	969,045
13. predict$.mp	1,864,406
14. or/7-13	11,534,473
15. 6 and 14	444
Result	444
